# Venous Thromboembolism Therapy with Edoxaban in Daily Care Patients: Results from the DRESDEN NOAC REGISTRY

**DOI:** 10.1055/a-2635-4840

**Published:** 2025-07-05

**Authors:** Luise Tittl, Christina Köhler, Sandra Marten, Christiane Naue, Jan Beyer-Westendorf

**Affiliations:** 1 Division Hematology, Department of Medicine I, Thrombosis Research Unit, University Hospital “Carl Gustav Carus” Dresden, Dresden, Germany

**Keywords:** anticoagulation, bleeding, persistence, edoxaban, venous thromboembolism

## Abstract

Direct oral anticoagulants such as edoxaban are standard of care in current treatment of venous thromboembolism (VTE). However, phase III trial data need confirmation in real-world settings.

We extracted data from the prospective, noninterventional multiple-indication DRESDEN NOAC REGISTRY to evaluate outcome rates during VTE treatment with edoxaban. Patients were included in this analysis, if they had acute VTE and if patient enrolment and edoxaban initiation occurred within 30 days after VTE diagnosis. Patient characteristics, treatment persistence, and clinical outcomes were centrally adjudicated using standard definitions.

Until December 31, 2023, 323 acute VTE patients (median age 67 years, 56.7% male) were enrolled and initiated edoxaban within 7.8 ± 4.9 days (mean) for isolated deep vein thrombosis (DVT) (57.6%) or pulmonary embolism (PE) ± DVT (42.4%). Mean duration of follow-up was 3.9 ± 1.9 years with a mean duration of edoxaban exposure of 1.5 ± 1.7 years. During ongoing edoxaban therapy, 3/323 patients (0.9%) experienced recurrent VTE (0.6/100 patient-years); 141/323 (43.7%) patients reported clinically relevant International Society on Thrombosis and Haemostasis (ISTH) nonmajor bleeding and 16 reported ISTH major bleeding (5.0%; 3.2/100 patient-years). Death was observed in 53 patients (4.1/100 patient-years). At 6 months, 78.2% were still taking edoxaban, 2% were electively switched to dose-reduced secondary prophylaxis with apixaban 2.5 mg twice a day or rivaroxaban 10 mg once daily. The remaining patients had a scheduled end of VTE treatment (11.4%) or were switched to nonedoxaban therapeutic anticoagulation (6.2%).

Our results indicate effectiveness of edoxaban in acute VTE treatment with excellent persistence in the treatment and low rates of unplanned discontinuation. Bleeding was frequently observed, but rates of major bleeding were low and comparable to phase III data.

## Introduction

During the last 15 years, direct oral anticoagulants (DOAC) such as edoxaban, have become the mainstay of venous thromboembolism (VTE) treatment. Besides high efficacy, the main benefits of DOAC include a predictable dose–response relationship, fixed dosing without a need for frequent monitoring and dose adjustments, and an acceptably low risk for major bleeding. 1 2 Within the class of DOAC, edoxaban is somewhat different from apixaban and rivaroxaban, as edoxaban has a much higher volume of distribution (∼110 vs. 50 and 21 L for rivaroxaban and apixaban, respectively) and a much higher renal excretion rate (50 vs. 33 and 27%, respectively). 3 4 5 Therefore, the risk of renal accumulation or rebound from tissue redistribution after treatment interruption could be, at least in theory, higher with edoxaban compared with apixaban or rivaroxaban. Consistent with this pharmacokinetic profile, edoxaban should only be used with caution in patients with high creatinine clearance, as a trend toward reduced antithrombotic efficacy was observed in this subgroup in atrial fibrillation trials. 3 Edoxaban has successfully completed large phase III trials in VTE treatment, including patients with cancer-associated thromboembolism. 6 7 However, patient selection treatment patterns and outcome event rates from randomized trials may differ from those observed in real-world settings. Therefore, a number of observational studies have evaluated routine care data for VTE treatment with apixaban and rivaroxaban. 8 9 10 11 12 13 For edoxaban, only the multicentric ETNA-VTE 14 registry has reported real-world outcomes in this setting, but here patient recruitment per site was low (on average only four enrolled patients per site per year, indicating potential for selection bias) and follow-up (FU) was limited to 3 months only. More studies providing long-term treatment data for VTE treatment with edoxaban are therefore needed.

With this in mind, we used data from the prospective multicentric cross-indication DRESDEN NOAC REGISTRY and report management patterns and outcome data for acute VTE management with edoxaban.

## Methods

### Patients

The DRESDEN NOAC REGISTRY (NCT01588119) is a prospective registry in the administrative district of Dresden (Saxony), Germany, enrolling DOAC-treated patients from a network of more than 230 physicians. No exclusion criteria apply. The registry design and methodology of evaluating VTE treatment with apixaban or rivaroxaban has been published previously, 9 10 and the current analysis for the edoxaban VTE cohort used the same approach. In short, all patients undergo prospective FU, based on standardized telephone visits performed by the central registry office. All patient-reported suspected outcome events are reviewed by a central adjudication committee, based on collected source documents.

For the presented analysis, only patients with acute PE and/or acute distal or proximal lower limb DVT who started edoxaban within 14 days after diagnosis of VTE and who were enrolled within these 14 days were evaluated with regard to patient characteristics, treatment persistence, and clinical outcomes.

The categorization of the index VTE event as provoked or unprovoked was performed according to American College of Clinical Pharmacy guidelines: 15

- VTE provoked by major surgery/major trauma within the past 3 months (a major transient risk factor).
- VTE provoked by a nonsurgical transient risk factor (e.g., estrogen therapy, pregnancy, nonfracture leg injury, flight of greater than 8 hours).
- Cancer-associated VTE (defined as cancer diagnosed within the previous 6 months; recurrent, regionally advanced, or metastatic cancer; cancer for which treatment had been administered within the previous 6 months; or hematologic cancer that was not in complete remission).
- Unprovoked VTE.

### Outcome Measures

Assessment of edoxaban effectiveness in VTE treatment was based on the annualized rate of the recurrent VTE, with cases of sudden death of unknown cause being adjudicated as fatal PE and counted as a recurrent VTE event.

Assessment of edoxaban safety was based on the annualized rate of major bleeding (main safety outcome) was according to the International Society on Thrombosis and Haemostasis (ISTH) definition, 16 with rates of ISTH clinically relevant nonmajor (CRNM) bleeding and all-cause mortality being secondary endpoints.

Crude outcome numbers are reported for days 90, 180, 365, and >365 and annualized event rates for 180 and 365 days.

### Treatment Discontinuation

In accordance with previously published analyses from our registry, edoxaban treatment discontinuation was defined as a permanent discontinuation or an unscheduled interruption for longer than 4 weeks without the initial plan to restart edoxaban, 9 10 17 which included patients who were permanently switched to another anticoagulant. In contrast, treatment persistence was defined as the continuation of edoxaban therapy over the entire FU period, allowing for any temporary interruption. In case of edoxaban discontinuation, reasons for switching to other anticoagulants or stopping anticoagulation as well as the future treatment plan were obtained from patients or attending physicians. Missing values were left blank and not replaced by imputation.

### Statistics

Two different analysis sets were defined and evaluated:

(1) The overall rate of recurrent VTE was evaluated in the intention-to-treat analysis, including all VTE patients who were enrolled in the registry and received edoxaban for acute VTE at baseline. In this analysis, all effectiveness outcome events were included that occurred throughout the FU period, including those occurring at any time during or after temporary interruption or discontinuation of edoxaban.
(2) Furthermore, rates of recurrent VTE events on treatment and rates of bleeding complications (all, major, and CRNM bleeding) were evaluated in the on-treatment analysis. This analysis also included all VTE patients enrolled in the edoxaban VTE cohort at baseline, but only outcome events that occurred during ongoing edoxaban treatment or within 3 days after the last intake (in case of temporary interruption or permanent discontinuation of treatment) were included.

Baseline characteristics are presented as absolute and relative frequencies, mean and standard deviation, or median with interquartile range as difference between 25th and 75th percentile, where appropriate.

In both the intention-to-treat and the on-treatment analysis set, outcome event rates were calculated using Kaplan–Meier time-to-first-event analysis, with data presented as events per 100 patient-years with their 95% confidence intervals (CI), using the following formula: Event rate = number of events / total time under risk (defined as the sum of all days from inclusion in the registry until the day of the first event divided by 100 × 365 days and 100 patient-years as its unit). Corresponding CI and *p* -values were calculated using the Poisson distribution.

Due to comparatively small sample sizes, any subgroup analyses are presented in a descriptive manner only and numerical differences were not assessed for statistical significance to avoid type 2 error.

All statistical analyses were carried out using the IBM SPSS Statistics version 25, MedCalc version 14.8.1.

### Ethics

The study protocol of the DRESDEN NOAC REGISTRY was approved by the local ethics committee at the Technical University Dresden (AZ EK 349092011) and registered at ClinicalTrials.gov (NCT01588119). The study complies with the principles and requirements of the Declaration of Helsinki. All patients provided written informed consent, including a data protection waiver, before enrolment.

## Results

**Fig. 1:**
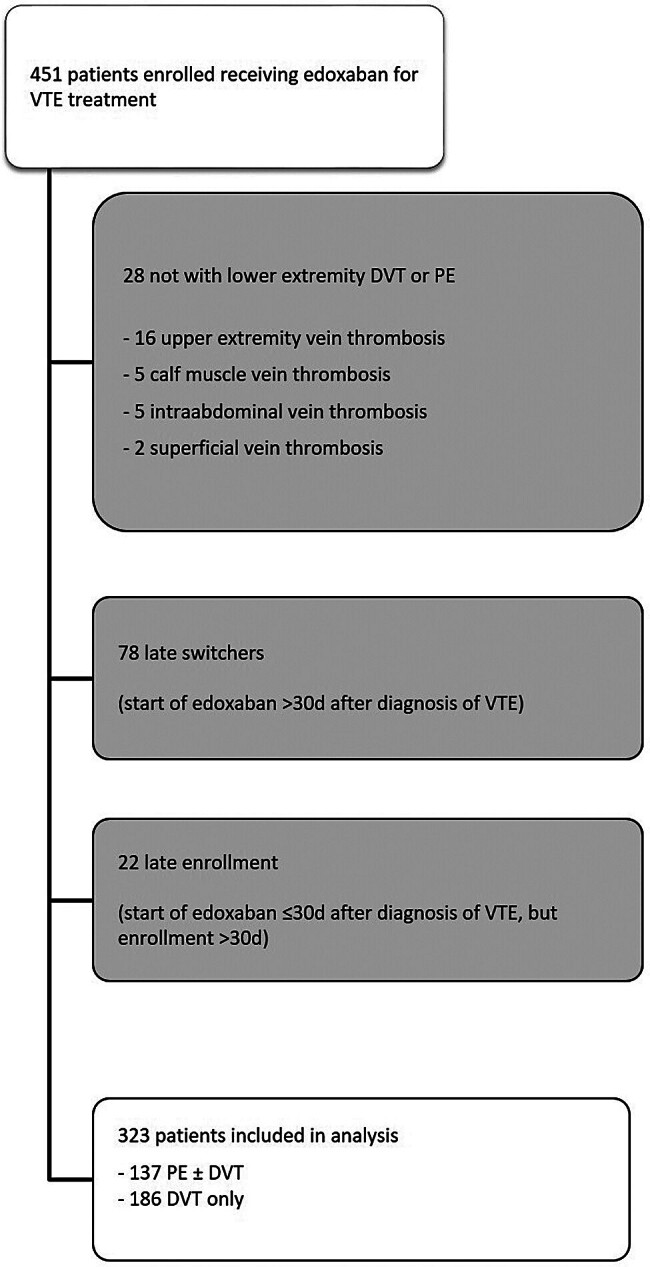
Flowchart of study cohort.

Between December 1, 2011 and December 31, 2023, a total of 5,252 patients were enrolled into the DRESDEN NOAC REGISTRY. Of these, 451 were receiving edoxaban for VTE treatment and 323 (71.6%) fulfilled the selection criteria for the present analysis. Reasons for exclusion are demonstrated in Fig. 1 .

Within the cohort of 323 evaluable patients, index VTE event was an isolated DVT without confirmed PE in 186 (57.6%) cases and 137 (42.4%) patients had an objectively confirmed PE with or without DVT. Overall, 56.7% were male and median age was 67 years (25th/75th percentile 54/77 years). Details on patient characteristics and index VTE are presented in Table 1 . Mean time between VTE diagnosis and initiation of edoxaban was 7.8 ± 4.9 days (median 6 days; 25th/75th percentile 5/9 days) and numerically longer for PE versus DVT (mean 8.2 ± 5.0 vs. 7.5 ± 4.8 days). At baseline, edoxaban was prescribed at a dose of 60 mg once daily in 83.9% and 30 mg once daily in 16.1% of patients.

**Table 1:** Patient characteristics at baseline

	All *n* = 323	DVT *n* = 186	PE ± DVT *n* = 137
Male, *n* (%)	183/323 (56.7)	98/186 (52.7)	85/137 (62)
Median Age (25 ^th^ /75th percentile)	67 (54–77)	68 (55–77)	66 (53–76)
Mean time between VTE diagnosis and initiation of edoxaban (SD)	7.8 ± 4.9	7.5 ± 4.8	8.2 ± 5.0
Unprovoked VTE, *n* (%)	193/323 (59.8)	117/186 (62.9)	76/137 (55.5)
Cancer-associated VTE, *n* (%)	39/323 (12.1)	14/186 (7.5)	25/137 (18.2)
Event VTE provoked by minor persistent or transient triggers, *n* (%)	29/323 (9)	21/186 (11.3)	8/137 (5.8)
Event VTE provoked by major transient triggers, *n* (%)	62/323 (19.2)	34/186 (18.3)	28/137 (20.4)
Recurrent VTE, *n* (%)	76/323 (23.5)	52/186 (28)	24/137 (17.5)
Proximal vs. distal DVT, *n* (%)	152/323 (47.1) 34/323 (10.5)	152/186 (81.7) 34/186 (18.3)	
Malignant disease, *n* (%) Active cancer, *n* (%)	85/323 (26.3) 31/323 (9.6)	39/186 (21) 11/186 (5.9)	46/137 (33.6) 20/137 (14.6)
Glomerular filtration rate (GFR) < 50 mL/min, *n* (%) GFR 30–50 mL/min, *n* (%) GFR < 30 mL/min, *n* (%)	41/323 (12.7) 33/323 (10.2) 8/323 (2.5)	28/186 (15.1) 25/186 (13.4) 3/186 (1.6)	13/137 (9.5) 8/137 (5.8) 5/137 (3.6)
Heart failure, *n* (%)	21/323 (6.5)	12/186 (6.5)	9/137 (6.6)
Arterial hypertension, *n* (%)	197/323 (61)	103/186 (55.4)	94/137 (68.6)
Diabetes mellitus, *n* (%)	37/323 (11.5)	21/186 (11.3)	16/137 (11.7)
Prior TIA, stroke, or systemic embolism, *n* (%)	13/323 (4)	5/186 (2.7)	8/137 (5.8)
PAD/CAD, *n* (%)	23/323 (7.1)	11/186 (5.9)	12/137 (8.8)

Abbreviations: DVT, deep vein thrombosis; IQR, interquartile range; PAD/CAD, peripheral arterial occlusive disease/coronary artery disease; SD, standard deviation; TIA, transient ischemic attack; VTE, venous thromboembolism.

### Edoxaban Effectiveness

**Fig. 2:**
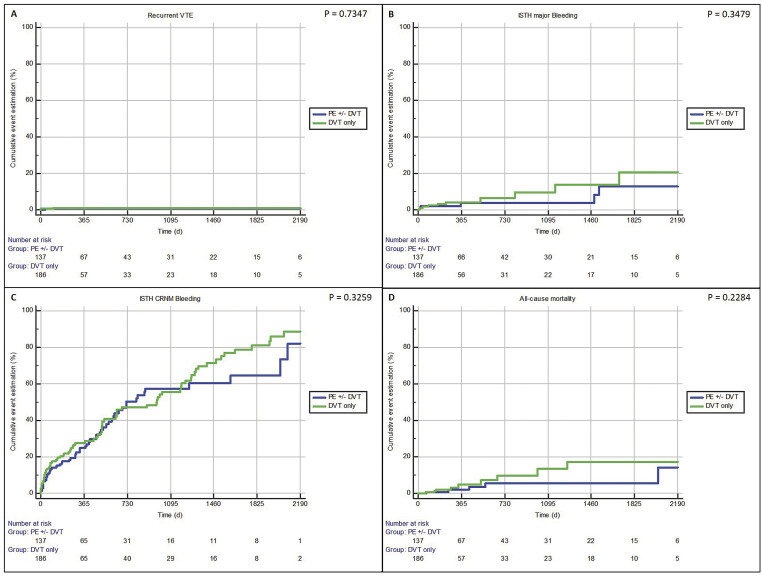
On-treatment outcome rates of DVT and PE treatment with edoxaban: VTE recurrence ( **A** ), ISTH major bleeding ( **B** ), CRNM bleeding, and ( **C** ) all-cause mortality ( **D** ). CRNM, clinically relevant nonmajor; DVT, deep vein thrombosis; ISTH, International Society on Thrombosis and Haemostasis; PE, pulmonary embolism; VTE, venous thromboembolism.

During a mean FU of 3.9 ± 1.9 years (median 4 years; 25th/75th percentile 2.4/5.5 years), a total of 28 patients (8.7%) experienced a recurrent VTE, which translated into a recurrence rate of 2.3/100 patient-years (95% CI: 1.6–3.4) for the intention-to-treat population ( Table 2 and Fig. 2 ).

**Table 2:** Outcome event rates according to treatment phase and treatment continuation

*n* = 323	Events at 90 d		Events at 180 d		Events at 365 d		Events > 365 d	
	ITT	On treatment	ITT	On treatment	ITT	On treatment	ITT	On treatment
Recurrent VTE, *n* (%)	4 (1.2)	2 (0.6)	3 (0.9)	1 (0.3)	3 (0.9)	0 (0)	18 (5.6)	0 (0)
Fatal VTE, *n* (%)	0 (0)	0 (0)	0 (0)	0 (0)	0 (0)	0 (0)	1 (0.3)	0 (0)
Major bleeding, *n* (%)		7 (2.2)		1 (0.3)		2 (0.6)		6 (1.9)
Fatal bleeding, *n* (%)		0 (0)		0 (0)		0 (0)		0 (0)
Mortality, *n* (%)	7 (2.2)	2 (0.6)	3 (0.9)	2 (0.6)	8 (2.5)	3 (0.9)	35 (10.8)	7 (2.2)

Abbreviations: ITT, intention-to-treat population, which includes all outcome events during follow-up, irrespective of anticoagulation status; VTE, venous thromboembolism.

The mean time of edoxaban exposure was 1.5 ± 1.7 years (median: 0.8 months; 25th/75th percentile 0.5/1.7 years) and, during active treatment with edoxaban, 3 patients experienced a recurrent VTE. This translated into a recurrence rate of 0.6/100 patient-years (95% CI: 0.1–1.8) for the on-treatment population. VTE recurrence rates were numerically higher in patients with DVT as an index event (1.0/100 patient-years; 95% CI: 0.1–3.6) compared with PE as an index event (0.5/100 patient-years; 95% CI: 0.0–2.8).

### Safety Outcomes

A total of 168 patients (52.0%; 84/100 patient-years; 95% CI: 71.8–97.7) reported a bleeding event of any severity during edoxaban exposure ( Table 2 , Fig. 2 ). ISTH major bleeding occurred in 16 cases (on-treatment rates 5.0% or 3.2/100 patient-years; 95% CI: 1.8–5.2), including 7 cases of gastrointestinal bleedings, 2 intracranial bleedings, 2 intraocular bleedings, 2 intra-articular bleedings, 1 case of genitourinary bleeding, 1 case of postoperative spinal epidural bleeding, and 1 case of postoperative bleeding of unknown bleeding site (manifesting as acute drop in hemoglobin). In addition, 141 cases (43.7%; 35.3/100 patient-years; 95% CI: 29.7–41.6) reported CRNM bleeding events, predominantly manifesting as skin/mucosal bleeding (52.5%), genitourinary bleeding (19.9%), gastrointestinal bleeding (18.4%), or other bleeding types (9.2%). Again, rates of major bleeding were numerically higher for DVT versus PE patients (5.0/100 patient-years for index DVT; 95% CI: 2.4–9.2 vs. 3.0/100 patient-years for index PE; 95% CI: 1.1–6.5).

During FU, a total of 53/323 patients died (16.4%; 4.1/100 patient-years; 95% CI: 3.1–5.3), of which 14 deaths occurred during or within 3 days after last intake of edoxaban (2.8/100 patient-years; 95% CI: 1.5–4.7; Fig. 2 ). Most common causes of death were terminal malignant disease ( *n* = 23), followed by sepsis/infection ( *n* = 18), fatal cardiovascular event ( *n* = 5), age-related death ( *n* = 1), or other causes ( *n* = 6). No fatal bleeding occurred.

### Persistence to Edoxaban Treatment

At 6 months (FU completed in 321 patients), 95.6% were still alive. Of these, 78.2% were still taking edoxaban, 2% were electively switched to dose-reduced secondary prophylaxis with apixaban 2.5 mg twice a day or rivaroxaban 10 mg once daily. The remaining patients had a scheduled end of VTE treatment (11.4%) or were switched to nonedoxaban therapeutic anticoagulation (6.2%).

At 12 months (FU completed in 319 patients), the corresponding figures were: 91.5% were alive, 42.5% were still taking edoxaban, 26.7% were switched to dose-reduced secondary prophylaxis (6.2% apixaban 2.5 mg twice a day; 20.5% rivaroxaban 10 mg once daily), 18.5% had scheduled end of VTE treatment, and 8.2% were switched to other anticoagulants. Therefore, the proportion of patients with unplanned complete edoxaban discontinuation at 6 and 12 months was 2.3 and 4.1%, respectively.

A total of 27 patients experienced a recurrent VTE (7 PE ± DVT, 20 DVT) off-treatment (edoxaban interruption >3d or permanent discontinuation), with a median time between the last intake of edoxaban and VTE recurrence of 5.3 months (25th/75th percentile 1.1–29.3 months; range: 9–1,510 days).

## Discussion

Our data contribute to the limited information on edoxaban effectiveness and safety in routine care VTE treatment. As stated before, ETNA-VTE 14 is the only large real-world study on VTE treatment with edoxaban, but its interpretation is limited by the large potential for selection bias and by the short FU period of 3 months only. As a consequence, more observational studies are needed to better understand patient selection, treatment patterns, and treatment effects of edoxaban in routine care.

In our cohort of prospectively followed VTE patients from a single county in Germany, we confirmed that patterns of patient selection, heparin pretreatment duration, and effectiveness and safety of edoxaban seen in phase III studies are very much generalizable to routine care. We observed that approximately 60% of selected patients had isolated DVT and 40% were treated for PE, with 23% being treated for recurrent VTE (distribution in HOKUSAI: 60% DVT, 40% PE, 19% with previous VTE). 6 In our study and in HOKUSAI, 57% of patients were male, although our cohort was of older age (mean: 67 vs. 56 years in HOKUSAI). Of note, ETNA-VTE Europe 18 reported comparable patient profiles (53% male, mean age: 65 years, 40% PE, 23% with recurrent VTE).

Median time between VTE diagnosis and edoxaban initiation was 6 days in our cohort and comparable to the observations in HOKUSAI (median: 7 days) and ETNA-VTE Europe (median: 6 days). Proportions of the first prescription of edoxaban 60 versus 30 mg/d in our cohort were 84 versus 16%, compared with 82 versus 18% in HOKUSAI and 88 versus 12% in ETNA-VTE Europe, respectively. Overall, we conclude that, apart from age, patient profiles and prescription patterns were in nearly full agreement with the patterns studied in HOKUSAI, underlining the external validity of this phase III trial.

At the same time, we found that patient characteristics somewhat differed from the baseline profiles of our apixaban and rivaroxaban VTE cohorts reported previously. 9 10 19 Patients in the current edoxaban cohort were more often male (57 vs. 51% in apixaban and 48% in rivaroxaban cohort) and more often had unprovoked VTE (60 vs. 33.5 vs. 21.8%, respectively). At the same time, more patients had a PE diagnosis (42 vs. 22 vs. 19%), which may be explained by the much higher proportion of patients with active cancer (10 vs. 1 vs. 3%), since cancer patients more often undergo chest imaging for cancer staging. These differences in patient profiles between our different DOC VTE cohorts are likely driven by a number of reasons: availability (edoxaban was approved in Germany 5 years after apixaban or rivaroxaban), learning curves and changing preferences of prescribers over time, pharmacological considerations (different risk for drug–drug interactions), and cost considerations (DOAC prices vary across Germany). It is therefore important to reflect the reported treatment outcomes according to the patient profile and to avoid cross-cohort comparisons, which is also a limitation for comparisons across different edoxaban studies.

Due to the short FU period in ETNA-VTE, comparisons of treatment outcomes were not feasible. In contrast, HOKUSIA allowed for individualized treatment durations between 3 and 12 months, but this also limits the interpretation and comparison of outcome data with our study. However, Hokusai reported on-treatment VTE recurrence rates of 1.6% with numerically higher rates for DVT (1.9) versus PE patients (1.1%), with overall patterns being confirmed in our cohort study (0.6, 1.0, and 0.5/100 patient-years; respectively).

Rates of major and CRNM bleeding were somewhat higher in our cohort. We observed on-treatment rates of 3.2 (major bleeding) and 35.3/100 patient-years (CRNM), whereas the corresponding rates were 1.4% (major bleeding) and 7.2% (CRNM) in HOKUSAI. We can only speculate if this difference was due to a reporting bias, different statistical evaluations or true treatment effects in a cohort that was nearly 10 years older than the phase III cohort. Furthermore, phase III anticoagulation trials often exclude patients at high risk for bleeding complications, whereas the DRESDEN NOAC REGISTRY does not apply any exclusion criteria. As such, patients' baseline bleeding risk may also have been different, causing higher bleeding rates in our observational study cohort.

Another important observation in our study was the very low rate of unscheduled premature discontinuation (2.3% at 6 and 4.1% at 12 months) and the clear trend toward prolonged anticoagulant treatment with edoxaban, since nearly 80% of patients were treated for longer than 6 months. The low rate of unscheduled premature discontinuation is reassuring, an indicator of high patient and physician satisfaction. The trend to prolonged treatment is in line with current guideline recommendations 20 21 22 and in line with previously reported VTE treatment cohorts with apixaban 9 and rivaroxaban. 10

### Limitations

There are several limitations to our study, which have been discussed in detail in our previous publications. 10 23 24 Our cohort consisted of 323 patients, and our sample size as well as the small number of outcome events limits the detection of clinically relevant treatment effects or subgroup analyses. As with all observational cohort studies, confounding effects from patient selection, from regional effects, underreporting of potential outcomes, and from the lack of randomized comparator need to be acknowledged. Still, postmarketing surveillance studies (PMSS) and observational registries also provide perspectives that randomized trials cannot evaluate. In phase III trials, patients at risk are often excluded; cohort profiles often optimized to show the desired treatment effects and dosing and patient management are strictly regulated by protocols. Furthermore, FU duration is often limited. Therefore, even within the limitations of our study design, the confirmation of phase III results in an older cohort observed over longer treatment and FU periods is an important addition to current literature and data collection at a patient level as well as central adjudication of all suspected outcome events are considerable strengths of our project.

## Conclusion

In daily care, edoxaban treatment for acute VTE is effective and acceptably safe. Patterns of patient selection, heparin pretreatment, edoxaban dosing, and treatment outcomes were strikingly comparable to phase III and PMSS data, supporting the external validity of these international studies in a regional prospective registry from a German county.

## Notes

**What is known about this topic?**

- Treatment of venous thromboembolism (VTE) with edoxaban was effective and safe in the HOKUSAI trials. However, due to the strict selection criteria in phase III trials, post marketing data are needed to confirm the generalizability of trial data.
- So far, real-world data on edoxaban treatment for acute VTE are limited, often derived from retrospective analyses and frequently lacking details on dosing, treatment persistence, or long-term outcomes.

**What does this paper add?**

- Data from ongoing, prospective, noninterventional Dresden NOAC Registry aimed to confirm the phase III trial results in daily care and to provide outcome data for up to 4 years.
- In daily care, acute venous thromboembolism (VTE) therapy with edoxaban showed acceptably low VTE recurrence and major bleeding rates over a follow-up duration of 4 years. Adherence to dosing recommendations and treatment persistence were satisfactory.
- Edoxaban demonstrated little differences in outcome rates for patients with isolated DVT or PE with or without DVT, confirming a broad generalizability of HOKUSAI trial data.
- VTE recurrence risk after stopping edoxaban was considerable, indicating a need for long-term outcome data.
